# Prohydrojasmonate–silicon synergy enhances cadmium detoxification and stress tolerance in rice, *Oryza sativa* L

**DOI:** 10.3389/fpls.2025.1731423

**Published:** 2026-01-19

**Authors:** Salem M. AL-Amri

**Affiliations:** College of Science and Humanities, Department of Biology, Shaqra University, Dawadmi, Saudi Arabia

**Keywords:** cadmium detoxification, phytoremediation, prohydrojasmonate, rice, silicon synergy

## Abstract

Cadmium (Cd) contamination severely threaten rice productivity and food security, yet effective and sustainable detoxification strategies remain limited. This study investigates whether combined application of prohydrojasmonate (PDJ) and silicon (Si) can synergistically enhance Cd detoxification in rice. Rice seedlings exposed to Cd stress were treated with PDJ, Si or their combination and evaluated through integrated physiological, biochemical and molecular analyses including metal accumulation, photosynthetic performance, oxidative status, hormonal regulation and gene expression. Compared to individual treatments, PDJ-Si co-treatment significantly reduced Cd translocation to aerial tissues, with maximum root retention restored essential leaf elements (Fe, K, Mn) and enhanced photosynthetic efficiency. While PDJ and Si individually enhanced membrane stability, reduced lipid peroxidation and improved osmotic balance their combined application produced the most pronounced effects. Phytohormone profiling revealed coordinated activation of salicylic acid (SA) and jasmonic acid (JA) pathways with balanced abscisic acid (ABA) modulation. Furthermore, both individual and combine application caused differential expression of genes related to detoxification (*OsABCC1*, *OsGSTU5*, *OsPCS1*), metal transporters (*OsHMA2*, *OsLCT1*) and hormone biosynthesis (*OsABA2*, *OsEDS1*, *OsAOS2*). Collectively, these findings demonstrate that PDJ and Si application enhance Cd detoxification and stress tolerance in rice providing a promising approach for sustainable rice cultivation in Cd-contaminated soils.

## Graphical Abstract

**Figure:**
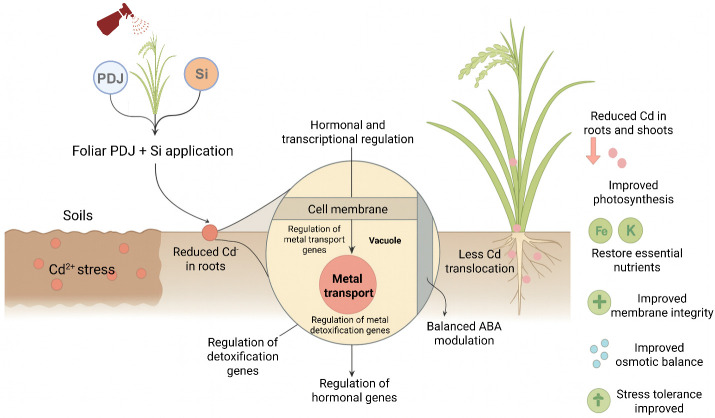
Foliar application of PDJ and Si synergistically mitigates Cd toxicity in rice by reducing Cd uptake and translocation while regulating metal transport genes, detoxification pathways and hormonal responses. The combined treatment enhances photosynthesis, restores nutrient balance, improves membrane integrity and osmotic balance, ultimately improving plant stress tolerance under Cd stress.

## 1 Introduction

Global food security faces unprecedented challenges due to the increasing contamination of agricultural soils with heavy metals (HMs), particularly cadmium (Cd), which threatens crop productivities and health concerns (Hou et al., 2025). Heavy-metals pollution represents a critical global environmental crisis, with Cd being one of the most toxic contaminants approximately 20% of China arable land is affected by HM contamination, with substantial areas across industrialized regions globally also experiencing severe pollution (Wen et al., 2022). Among food crops, rice (*Oryza sativa* L.) is vital staple-foods for around half of world populations, providing essential calories and nutrients to over 3.5 billion peoples (Seck et al., 2012). However, rice cultivation faces significant threats from various abiotic stresses, with HMs contamination, particularly Cd toxicity, emerging as a critical concern for both crop productivity and consumer safety (Peera Sheikh Kulsum et al., 2023). Rice is especially vulnerable to Cd accumulation, under flooded conditions, the activity of Cd in the soil layer is altered, resulting in the enhanced absorption and accumulation of Cd by rice (Dong et al., 2023). The capacity of rice to accumulate Cd at concentrations far exceeding regulatory safety limits presents a dual challenge compromised plant growth and development alongside serious human health risks through dietary consumption, as Cd is a known carcinogen and can impair kidney, bone and reproductive functions (Li et al., 2017; Yang et al., 2025).

Cd toxicity in rice manifest through multiple physiological and biochemical mechanisms. At the cellular level, Cd disrupts vital processes by displacing essential metal ions, inactivating enzymes and generating excessive reactive oxygen species (ROS) that harm cellular component including membrane, DNAs and protein (Mostofa et al., 2015; Yang et al., 2024). Furthermore, Cd-stress induces transcriptional reprogramming, alters phytohormone homeostasis and disrupts nutrient uptake mechanisms, collectively compromising the plant-growths and stress response capabilities (Song et al., 2024). Researcher are working to develop plant-based strategies that enhance Cd stress tolerance and minimize grain Cd accumulation while maintaining yield and quality parameters (Wang et al., 2019a). Several approaches have shown promise in mitigating Cd stress in rice. Agronomic and chemical mitigation approaches have shown varying degree of effectiveness in controlling Cd mobility and uptake. Agronomic practices including water management, soil amendments and fertilization regimes can reduce Cd bioavailability (Chen et al., 2023; Jing et al., 2021). More recently, the foliar applications of beneficial elements, phytohormones, signaling molecules and antioxidants have emerged as practical and economical strategies to increase plant resilience against Cd stresses (Chen et al., 2021; Kaur et al., 2023; Peng et al., 2023). Among these, combined or synergistic treatment approaches have gained increasing attention, as they may provide better protection compared to single-agent applications (Khalequzzaman et al., 2024; Khan et al., 2021; Oliveira et al., 2020; Shohani and Fazeli, 2025).

Silicon (Si) stands out among beneficial elements for its remarkable capacity to relieve numerous biotic or abiotic stress in plant, particularly in rice—a well-documented Si-accumulating species (Liang et al., 2015). The second most-abundant among elements in the Earth crusts, Si offers an economically viable and environmentally sustainable option for stress mitigation (Grewal et al., 2024). Following Cd exposures, Si supplementation has been revealed to strengthen physical barriers through silica deposition in cell walls, complex with Cd in the apoplast, stimulate antioxidant defense systems, regulate transporter gene expression and maintain essential nutrient homeostasis (Cui et al., 2017; Farooq et al., 2016). Specifically, Si induced formation of complexes in cell walls prevent Cd translocation to the shoots, while Si simultaneously activate the expression of antioxidant enzymes such as superoxide dismutase, catalase and peroxidase reducing ROS induced cellular damage (Liang et al., 2003). Despite these benefits, Si applications alone might not provide comprehensive protection against severe Cd stress, particularly under prolonged exposure or high contamination levels (Zargar et al., 2019). These limitations indicate that combining Si with other protective agents that target complementary stress-response pathways may improve overall Cd tolerance.

Phytohormones, play key role in plants health and stress response, hold strong potential to act synergistically with Si in improving crop-tolerance to HMs stress (Saini et al., 2021; Shafqat et al., 2024). Jasmonates, particularly jasmonic acid (JA) and his derivative like methyl jasmonate (MeJA), play important role in plant health and defense response against both biotic as well abiotic stresses (Hewedy et al., 2023; Wang et al., 2021). JA and its derivatives regulate the expression of genes encoding defensive secondary metabolites and antioxidant enzymes through the jasmonate JAZ-mediated signaling pathway, making them important regulators of plant immunity and stress resilience (Zhao et al., 2024). However, the challenges associated with the stability and field applicability of JA and its derivative MeJA can be compromised under certain environmental conditions (Bhavanam and Stout, 2021; Dorado et al., 2025; Riemann et al., 2015).

Prohydrojasmonate (PDJ), a synthetic JA analogue developed as plant growth regulator, has gained attention for its enhanced stability and biological activity compared to natural jasmonates (Tang et al., 2022). Unlike JA and MeJA, which are volatile and subject to faster degradation PDJ may enzymatically converted to JA only when needed by the plant, allowing for sustained and controlled defense responses while maintaining better field stabilities. Recent findings revealed that PDJ treatment elicit plant defense and induce the productions of several secondary metabolites (SMs) like anthocyanins, terpenoid, glucosinolates and phenolics (Azis et al., 2020; Takahashi et al., 2021). These SMs themselves possess antioxidant and chelating properties, which could contribute to HMs tolerance. Several findings indicate that PDJ can enhance many aspects of crop qualities. For instance, PDJ application recover the hand-picking efficacy of satsumas mandarins (Sato and Ikoma, 2016). In brassica, PDJ application altered the settling and behavioral response of cabbage aphids, *Brevicoryne brassicae* L (Ali et al., 2024). In lettuce and konatsuna, PDJ application known to induce phenolic compounds as well as anthocyanin induction without adverse effects on plants growth (Azis et al., 2020). In lettuce and konatsuna, PDJ application known to induce phenolic compounds as well as anthocyanin induction without adverse effects on plants growth (Azis et al., 2020; Yoshida et al., 2021).

However, some research has indicated that while PDJ enhances plant defense responses, it may also have opposing effects on crops growth, including potential trade-offs between defense investment and growth performances (Azis et al., 2020). These adverse effects of PDJ can be context dependent, such as inadequate dosage, excessive concertation, or specific environmental conditions. This apparent paradox necessitates careful evaluation of PDJ net effects on crops performance when applied to HMs-stresses system. While Si and jasmonates present two different protective strategies, their potential synergistic interaction in mitigating Cd stress in rice remain largely unexplored (Khalequzzaman et al., 2024; Rawat et al., 2023). Si largely operates through physical and biochemical mechanisms, reinforcing cell walls barriers, sequestering Cd in the apoplast and improving nutrient homeostasis (Pandey et al., 2025), whereas jasmonates such as PDJ activate signaling cascade that modulate gene expression, influence antioxidant enzymes system as well as induce the synthesis of protective SMs (Takahashi et al., 2021). These complementary mechanisms, physical-chemical defenses versus signal-mediated physiological response suggest that their combined treatment may provide improved and multifaceted protection against Cd toxicity comparison to individual–agent treatment (Oliveira et al., 2020; Rawat et al., 2023). To date, no studies have systematically examined the combined effects of Si and PDJ on Cd contaminated rice, nor have they evaluated whether their interactions produce additive, synergistic or antagonistic outcomes in terms of plant physiological performance.

Therefore, this study aims to systematically investigate the combined effects of Si and PDJ in enhancing Cd stress tolerance in rice, with particular emphasis on the restoration of photosynthetic performance and the underlying mechanisms of action. Our study clarifies the individual and interactive roles of these two protective agents and provided evidence based strategies for developing more effective approaches to mitigate Cd toxicity in rice production system.

## 2 Materials and methods

### 2.1 Plant culture

Rice (*Oryza sativa* L.) seeds were cleansed with distilled water, submerged briefly and held at 30 °C for one day in a moist, light-free environment. Emerged sprouts were positioned in compact trays (30 × 20 cm) filled with a nutrient solution for rice growth. These trays resided in a regulated enclosure at 27 ± 1 °C, illuminated for 16 hours daily. When seedlings attained a height of 5–6 cm, they were relocated to larger containers (40 × 30 × 10 cm) containing nutrient liquid, kept under identical warmth and light settings with a light intensity of 600–650 μmol m^−^² s^−^¹. Plants were anchored in container lid openings using foam supports. The nutrient liquid was oxygenated continuously via an aquarium pump to prevent oxygen scarcity. The liquid pH was set to 5.0 twice each day using 1N KOH or HCl and refreshed entirely every three days.

### 2.2 Experimental design and treatments

Experiment were performed using 21-day-old rice seedlings. On day 0, Cd stress was initiated by adding 100 μM CdCl_2_ (Shanghai Aladdin Biochemical Technology Co., Ltd., Shanghai, China) to the nutrients solution (Chen et al., 2019). Twenty-four hours after stress onset, seedlings were treated with a foliar spray of either 2000-fold diluted PDJ (5% PDJ, Aladdin) (Morino et al., 2022), 2.5 mM Si (Na_2_SiO_3_·9H_2_O) (Gao et al., 2018), or a combined PDJ+Si treatment. Follow-up sprays were applied on days 7 and 14, with the Cd stress maintained for 21 days total. Each spray solution contained 0.1% Tween-80 to improve adhesion and absorption. Treatment groups included: control (CK, sprayed with distilled water), PDJ, Si and PDJ+Si.

### 2.3 Cd accumulation and leaf elemental analysis

Cd content in rice seedling (leaves, stems, roots) were measured. Collected tissues were dehydrated at 80°C and grounded into a fine-powder. A 0.5 g portion of powder was broken down in a 4:1 (v/v) mix of HNO_3_ and H_2_O_2_. The digest was diluted to 25 mL, passed through Whatman filter sheets and examined for Cd using an ICP-MS (Thermo Fisher Scientific). Other minerals (Fe, Zn, Mn, Cu, Mg, Ca, K) were quantified in shoot tissues following the same protocol described above.

### 2.4 Plant biomass measurements

Post-harvest, plants were split into roots and shoots and their fresh masses were recorded instantly. Sample were then oven dried at 80°C until mass stabilized and dry masses were measured. Five plants per group were randomly chosen for these assessments.

### 2.5 Measurement of gas exchange activities and chlorophyll content

Photosynthetic traits, including CO_2_ assimilation, water loss rate and stomatal aperture, were evaluated with a portable LI-6400XT gas analyzer (USA). Three mature leaves per plant from each group were tested in a slim chamber with 1000 μmol m^−^² s^−^¹ light and 500 μmol s^−^¹ flow. Conditions were set at 400 μmol CO_2_ mol^−^¹ air and 2.0 kPa vapor pressure. Chlorophyll level were measured with Solarbio Chlorophyll Assay Kit (Beijing, China) per the provided guide. Fresh leaves were cleaned, dried and chopped finely. A 0.2 g sample was pulverized in one mL water in darkness, diluted to 10 mL in a flask and mixed well. After 3 hours in darkness, the liquid absorbance was checked at 660 nm with a Genesys 10 Bio spectrophotometer (Thermo Fisher Scientific).

### 2.6 Measurement of plant water relations and stress indicators

Leaf water retention was calculated by cutting discs from mature leaves. Initial mass (FM) was recorded, followed by soaking in pure water for 4 hours at ambient temperature to get saturated mass (SM). Discs were dried at 80°C for one day to find dry mass (DM). Water retention was computed as: (%) = [(FM - DM)/(SM - DM)] × 100. Membrane leakage was tested using 0.5 g of leaf segments (1 cm) in 10 mL deionized water. After one day at room temperature, baseline conductivity (C_1_) was measured with an EC112 meter (Thermo Scientific). Samples were then sterilized at 120°C for 20 minute, cooled and final conductivity (C_2_) was measured. Leakage was: (%) = (C_1_/C_2_) × 100. Malondialdehyde (MDA) was quantified by grinding 0.4 g leaves in 10 mL 0.1% trichloroacetic acid, then spinning down. The clear liquid was diluted with 0.5% TBA in 20% TCA, heated at 92°C for 29 minutes, cooled fast and spun again. Absorbance at 530 nm and 600 nm was used to calculate MDA with a 155 mM^−^¹ cm^−^¹ coefficient, reported as nmol g^−^¹ fresh mass. Proline was measured by crushing 0.6 g leaves in 3% sulfosalicylic acid, then filtering. The liquid was heated with acetic acid and acid-ninhydrin at 100°C for 1 hour, extracted with toluene and absorbance at 515 nm was compared to a standard curve, expressed as μg g^−^¹ fresh mass.

### 2.7 Phytohormone analysis

Rice leaves were frozen in liquid nitrogen and kept at -80°C. About 150 mg of frozen leaf was ground in liquid-nitrogen. A 1.5 mL ethyl acetate solution with internal standards (D_6_-JA, D_4_-SA, D_6_-ABA) was added. Samples were shaken in a GenoGrinder at 250 speed for 1.5 minutes, swirled for 5 minutes and chilled on ice for 1-hour. After spinning at 13500 rpm for 20 minutes at 4°C, the clear liquid was moved to a 2-mL tube. Extraction was repeated with 500 μL ethyl acetate and liquids were combined. The extract was dried at 30°C in a vacuum concentrator, dissolved in 500 μL 70% methanol, swirled for 5 minute and spun at 13500 rpm for 10 minute. A 400 μL portion was placed in an HPLC vial. Hormones (ABA, SA, JA) were measured with a Quattro Premier LC-MS/MS, using an Agilent 6460 mass spectrometer in negative ion MRM mode, separated on a Zorbax SB-C18 column (150 × 2.1 mm, 3.5 μm). Levels were based on internal standard peak areas (Forcat et al., 2008).

### 2.8 Confocal microscopy

Leaf segments (5–10 mm) from treated rice plants were carefully cut using a razor blade and promptly placed on glass slides in distilled water, covered with a coverslip to prevent air bubble formation. Imaging was conducted using a Zeiss LSM confocal laser scanning microscope. Chloroplasts were visualized by exciting chlorophyll auto-fluorescence at 488 nm, with emission captured between 650 and 750 nm. Cell walls were imaged by exciting auto-fluorescence at 405 nm, with emission detected from 420 to 480 nm. Fluorescence intensity and structural features were analyzed using Zeiss ZEN software.

### 2.9 Gene expression analysis

Total RNA was isolated from 100 mg rice tissue using TRIzol (Thermo Fisher Scientific). RNA concentration was checked using a NanoDrop 2000 (Thermo Fisher Scientific). cDNA was synthesized using ReverTra-Ace qPCR RT Master-Mix following gDNA Remover (TOYOBO, Japan). RT-PCR was run on a BioRad CFX96 system. Each 20-μL reaction had 2 μL cDNA, 10 μL SYBR-Green mix, 6 μL pure water and 1μL primers. The program started at 94°C for 3 minute, then ran 45 cycles of 94°C for 12 second and 65°C for 35 second. Primer specificity was confirmed with melt curves. *OsACTIN* (forward: *GTCCTCTTCCAGCCTTCCTT*; reverse: *CAATGCCAGGGAACATAGT*) normalized cDNA amounts. The list of primers for the investigated genes is provided in **Supplementary Table S1**.

### 2.10 Statistical analysis

Data were processed with one-way ANOVAs and treatment difference were identified using Tukey’s HSD at *p*<0.05. Pearson correlation coefficients were calculated among measured parameters using R software (method = “pearson”, use = “complete.obs”) to assess linear relationship between variable. Correlation matrices were visualized using corrplot package with circle-based plots showing the upper triangular matrix, where red and blue circles represent negative and positive correlations, respectively, with intensities proportional to correlation strength. Figures were drawn in OriginPro (2025), with statistical analysis performed in SPSS (29.0) and R (4.4.1).

## 3 Results

### 3.1 Cd accumulation and growth responses in rice under stress and mitigation treatments

**Figure 1:**
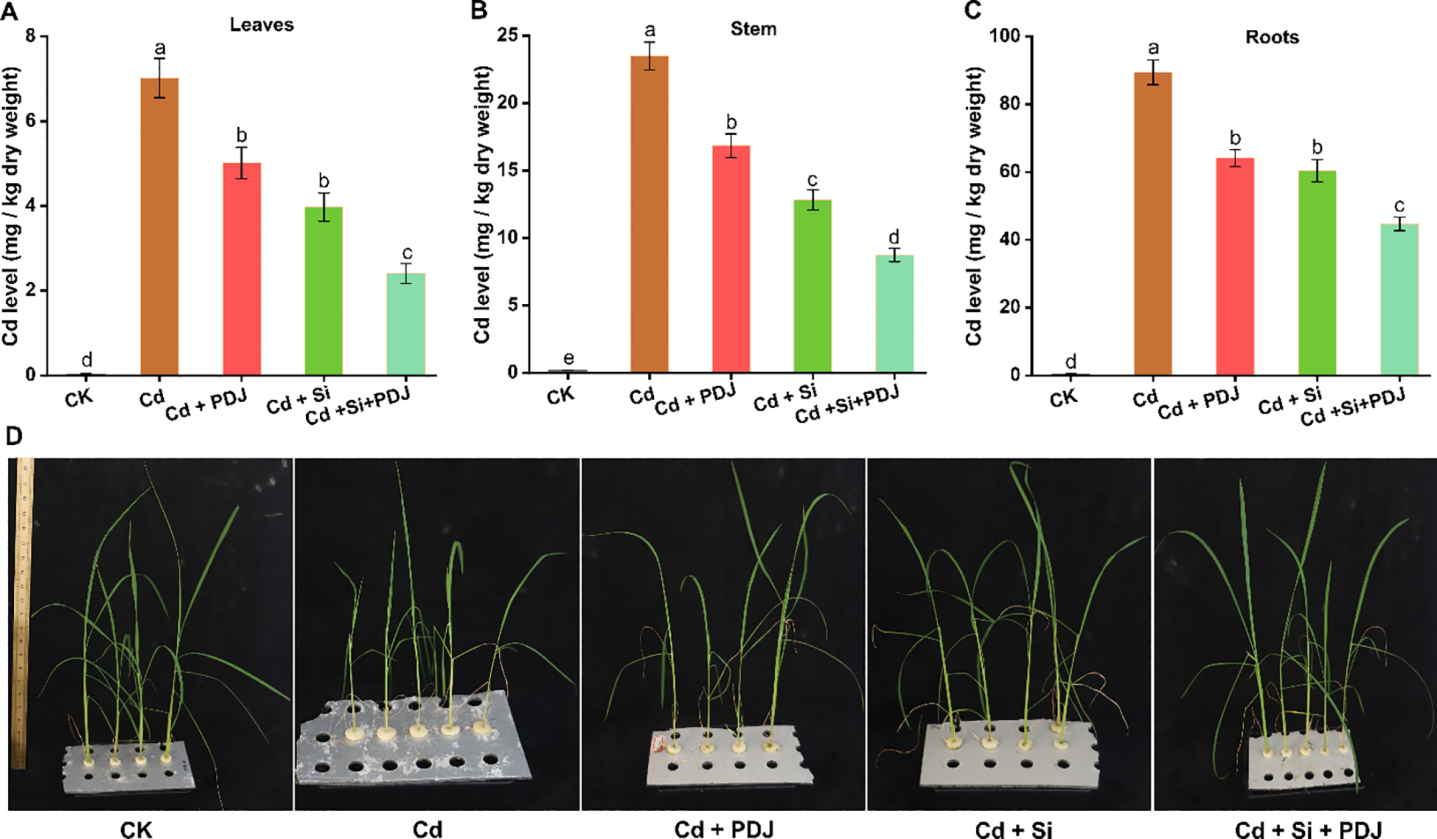
Cadmium accumulation in different tissue of rice plants under various treatment conditions. **(A)** Cd levels in leaves, **(B)** Cd levels in stem and **(C)** Cd levels in roots. **(D)** Representative rice plants under different treatments. Data are presented as mean ± standard error (*n* = 5 tests/treatment). Different letters above bars indicate statistically significant difference between treatments (*p* < 0.05) using ANOVAs followed by *post-hoc* analysis.

Cd accumulation exhibited distinct tissue-specific patterns with root tissues showing the highest concentration followed by stems and leaves under Cd stress conditions. This distribution pattern indicates preferential Cd sequestration in root tissues with limited translocation to aerial plant parts (**Figures 1A-D**). In leaves, PDJ application effectively reduced Cd accumulation, while Si supplementation achieved a 44% reduction compared to Cd treatment alone (F_4_,_24_ = 67.26, *p* < 0.001). The combined application demonstrated superior efficacy, reducing leaf Cd content by 67% (**Figure 1A**). Stem tissues exhibited similar protective responses, with PDJ and Si individually reducing Cd accumulation by 27% and 44% respectively, relative to Cd stress (*F_4_,_24_* = 145.2, *p* < 0.001). The synergistic treatment achieved a 63% reduction in stem Cd content (**Figure 1B**). Root Cd accumulation was moderately reduced by PDJ (31% reduction) and Si (36% reduction) treatments (*F_4_,_24_* = 157.4, *p* < 0.001). The combined treatment produced a 51% reduction in root Cd content, demonstrating enhanced protective effects through synergistic interactions between PDJ and Si across all plant tissues (**Figure 1C**).

**Figure 2:**
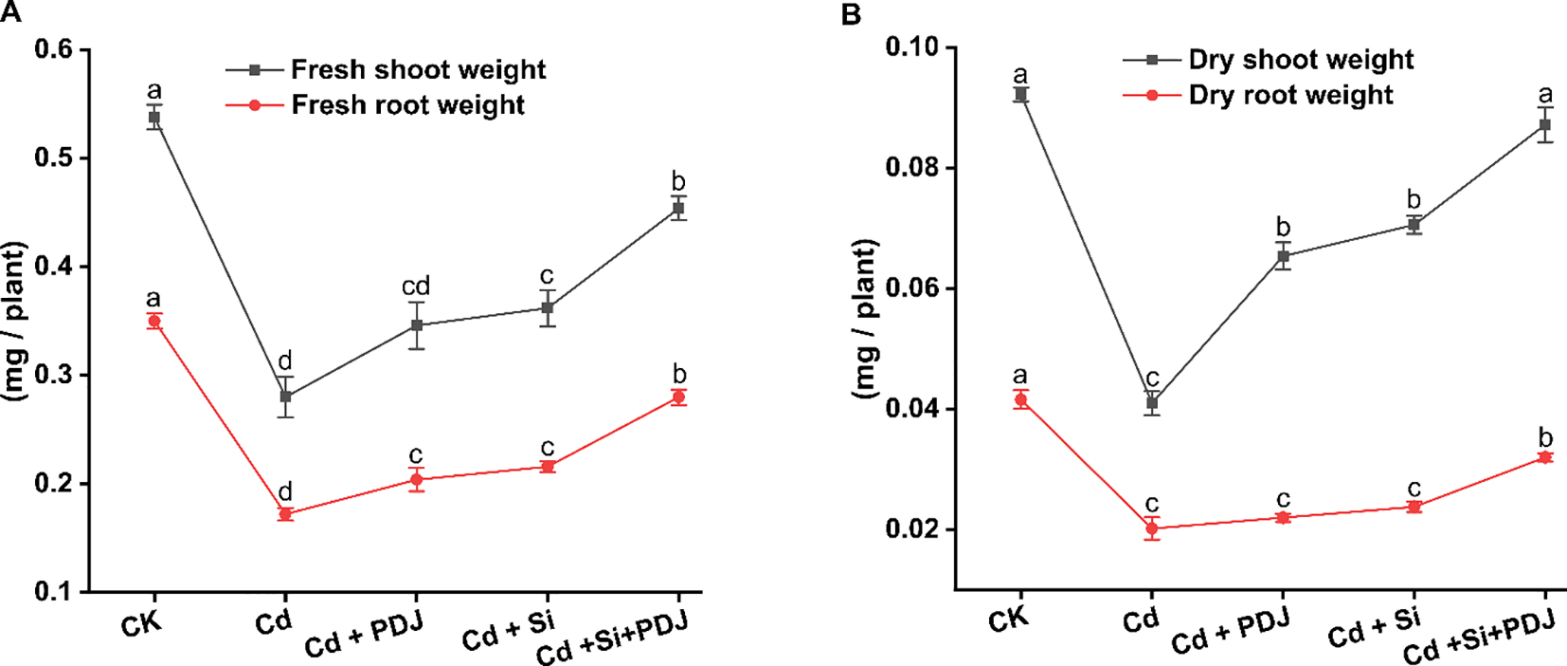
Effects of Cd exposure and mitigation strategies on rice seedling growth parameters. **(A)** Shoot and root fresh biomass measurements and **(B)** shoot and root dry biomass measurements for various treatment conditions. Data points show average values ± standard error (*n* = 5 tests/treatment). Different letters above columns indicate statistically significant difference among treatments based on Tukey’s honestly significant difference analysis (*p* < 0.05).

Furthermore, Cd stress severely affected plant growth and reduced plant biomass accumulation compared to control conditions (**Figure 2**). Fresh shoot weight declined under Cd stress (*F_4_,_24_* = 37.91, *p* < 0.001), while fresh root weight was decreased (*F_4_,_24_* = 91.02, *p* < 0.001) (**Figure 2A**). Similar pattern was observed for dry biomass, with shoot (*F_4_,_24_* = 99.21, *p* < 0.001) and root (*F_4_,_24_* = 52.99, *p* < 0.001) dry weights decreasing by 57% and 50%, respectively, under Cd treatment (**Figure 2B**). Both PDJ and Si treatments individually restored growth parameters, with Si showing superior recovery effects. The combined treatment (Si+PDJ) achieved significant restoration of fresh shoot weight and higher recovery of root biomass, demonstrating synergistic growth-promoting effects under Cd stress conditions.

### 3.2 Leaf elemental composition and photosynthetic responses

**Figure 3:**
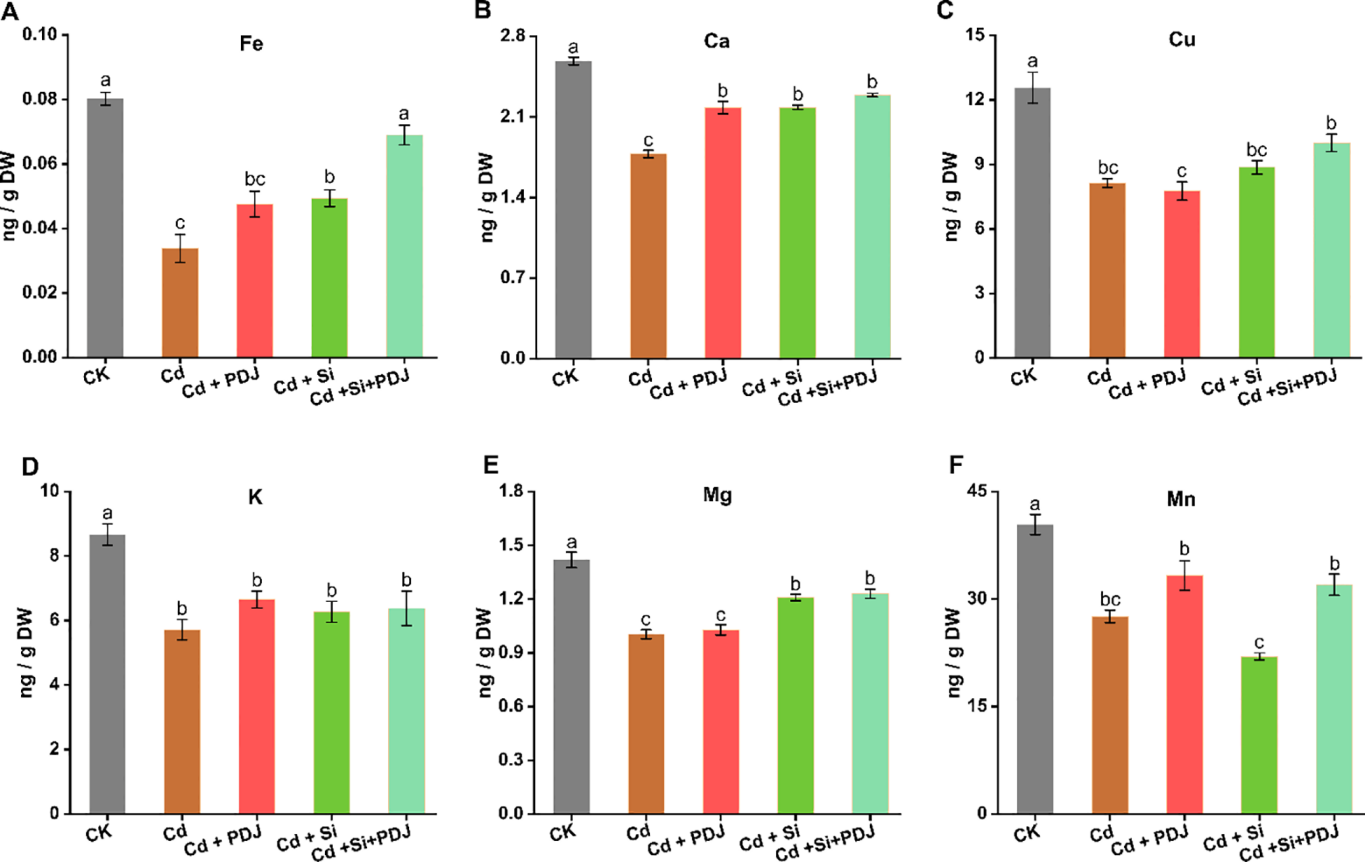
Effect of Cd exposure and remediation approaches on elemental content in rice shoot tissue. Distribution of essential minerals: **(A)** Iron, **(B)** Calcium, **(C)** Copper, **(D)** Potassium, **(E)** Magnesium and **(F)** Manganese levels within aerial plant parts under different treatment conditions. Data are presented as mean ± standard error (*n* = 5 tests/treatment). Different letters above bars indicate statistically significant difference between treatments (*p* < 0.05) using ANOVAs followed by *post-hoc* analysis.

Cd exposure severely disrupted leaf elements homeostasis across all examined elements, with reductions ranging from 22% to 58% compared to control conditions (**Figure 3**). Fe showed the most pronounced decline (58% reduction;*F_4_,_24_* = 31.75, *p* < 0.001), followed by K (*F_4_,_24_* = 9.55, *p* < 0.001) and Mn (*F_4_,_24_* = 25.15, *p* < 0.001) (both 35% reduction), Ca (*F_4_,_24_* = 76.76, *p* < 0.001) and Mg (*F_4_,_24_* = 33.15, *p* < 0.001) (32-33% reduction) and Cu (*F_4_,_24_* = 17.73, *p* < 0.001) (22% reduction) (**Figures 3A-F**). This systematic depletion indicates widespread interference with leaf elements uptake and transport mechanisms under Cd stress. Individual mitigation treatments demonstrated differential recovery patterns across leaf elements. PDJ application achieved moderate restoration for most elements, with recovery rates ranging from 25-40% of the Cd-induced losses. Si supplementation generally showed superior individual efficacy, particularly for Mg and Cu restoration (**Figures 3C, E**), achieving 35-45% recovery compared to Cd alone. Notably, both treatments achieved comparable and substantial recovery for Ca (**Figure 3B**), restoring concentrations in higher levels. The combined treatments of PDJ and Si consistently demonstrated optimal mineral restoration across all nutrients, achieving 70-90% recovery compared to control levels and significantly outperforming individual treatments for Fe, Cu and Mn (**Figures 3A, C, F**).

**Figure 4:**
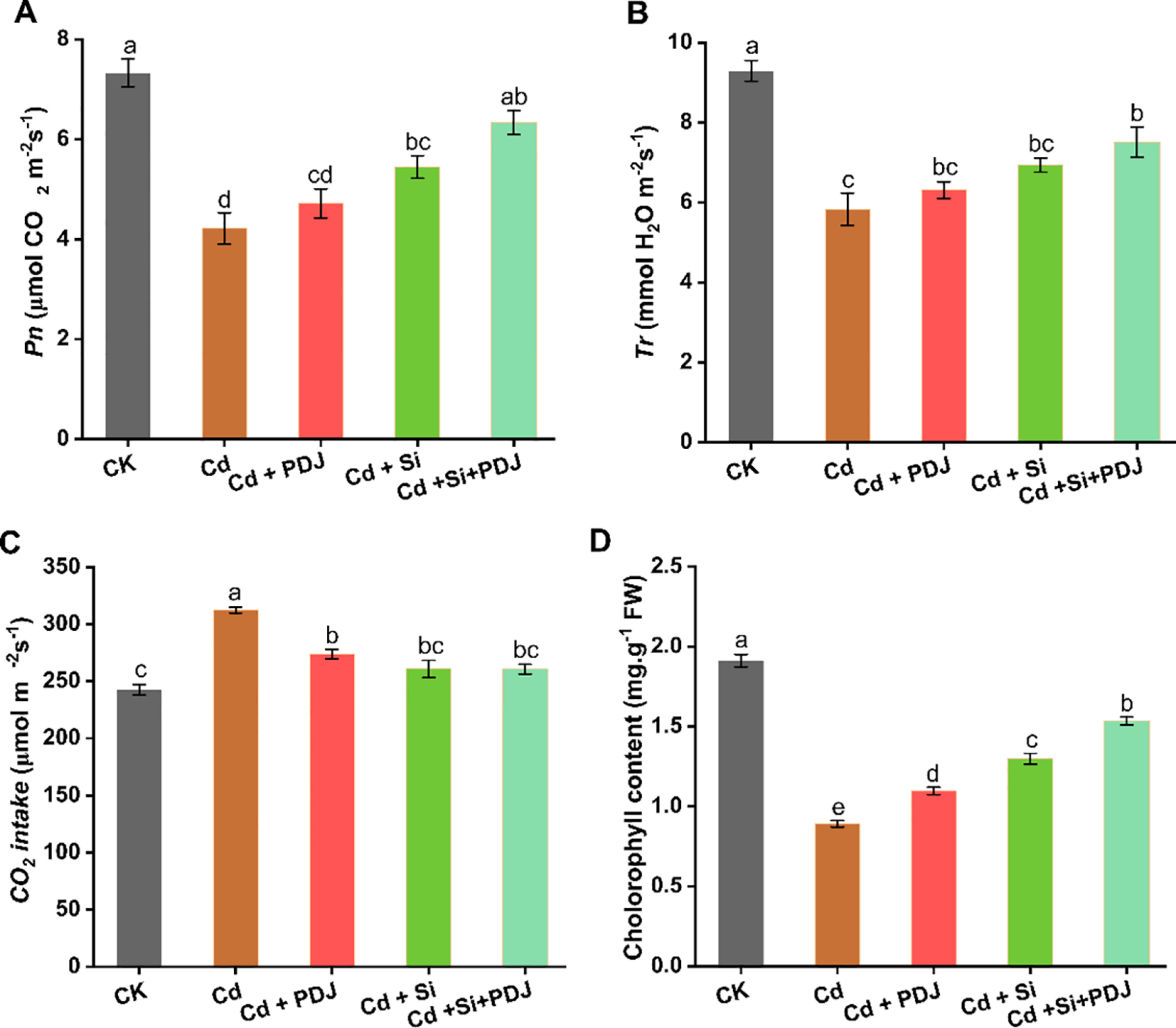
Effects of Cd exposure and mitigation strategies on photosynthetic performance. Physiological parameters and chlorophyll measurements: **(A)** Carbon assimilation rate, **(B)** Water vapor loss rate, **(C)** Stomatal aperture regulation and **(D)** Chlorophyll content levels under various treatment conditions. Data are presented as mean ± standard error (*n* = 6 tests/treatment). Different letters above bars indicate statistically significant difference between treatments (*p* < 0.05) using ANOVAs followed by *post-hoc* analysis.

**Figure 5:**
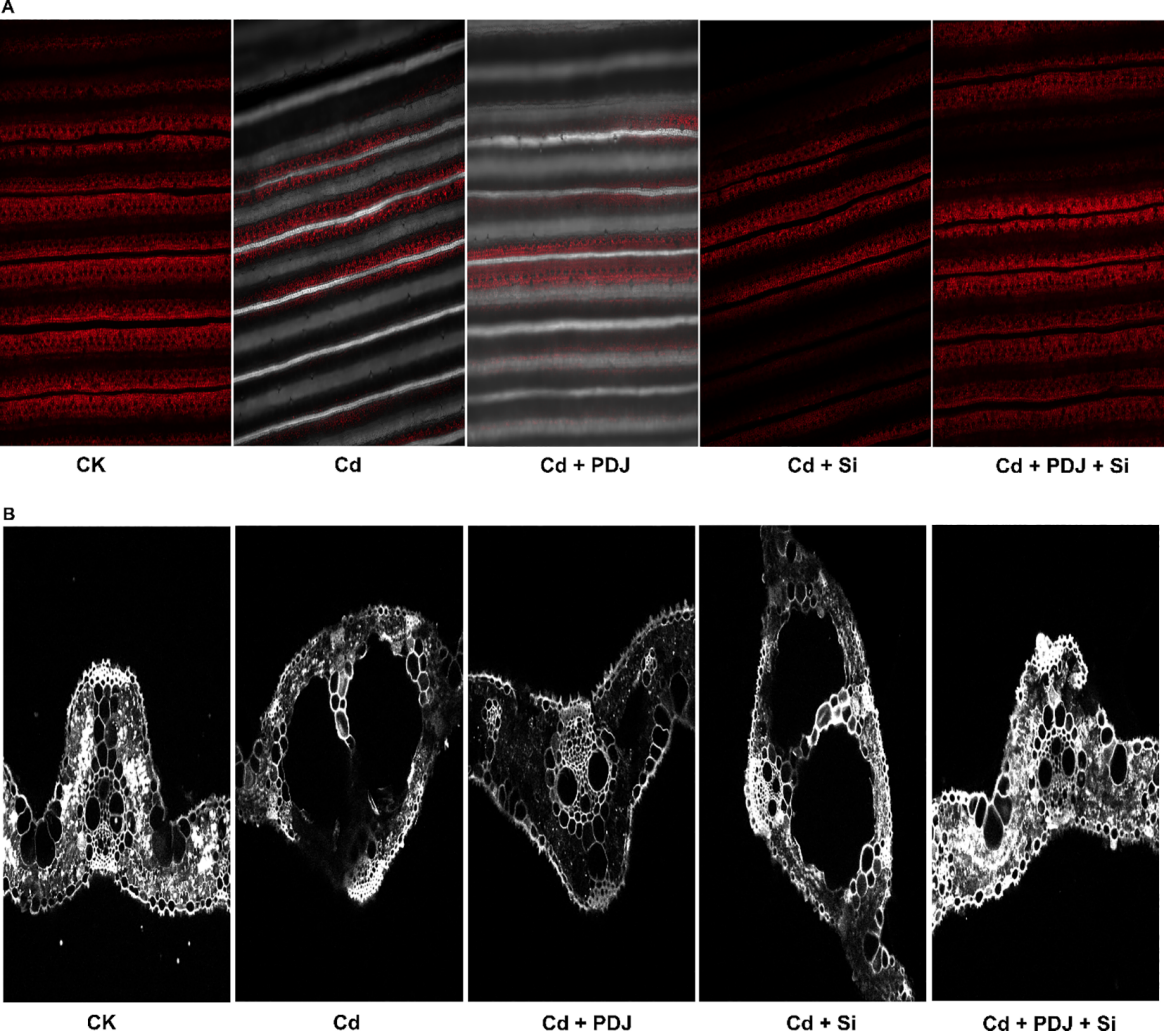
Effect of Cd stress and protective treatments on chloroplast organization and cell wall structure in mesophyll cells. **(A)** Upper panel showing chloroplast distribution (red fluorescence) in mesophyll cells. **(B)** Lower panel showing cell wall visualization in mesophyll cells.

Photosynthetic function was severely impaired by Cd exposure, with all measured parameters showing significant reductions except CO_2_ intake compared to control conditions (**Figure 4**). Net photosynthetic rate declined dramatically under Cd stress, representing a 43% reduction (*F_4_,_29_* = 21.09, *p* < 0.001) (**Figure 4A**). Similarly, transpiration rate showed 41% decrease indicating a decline in water vapor exchange (*F_4_,_29_* = 20.24, *p* < 0.001) (**Figure 4B**). CO_2_ uptake capacity was substantially increased under Cd treatment, representing a paradoxical increase that may reflect stress-induced metabolic disruption (*F_4_,_29_* = 28.38, *p* < 0.001) (**Figure 4C**). Chlorophyll content showed the most severe impact, declining by 53% under Cd stress (F_4_,_29_ = 188.06, *p* < 0.001), indicating significant damage to the photosynthetic apparatus (**Figure 4D**). Mitigation treatments demonstrated varying degrees of photosynthetic recovery. PDJ application moderately improved net photosynthetic rate and transpiration rate, while achieving partial chlorophyll restoration. Si treatment showed superior individual efficacy, restoring photosynthetic rate, transpiration and chlorophyll content. The combined treatment of PDJ and Si achieved optimal photosynthetic recovery across all parameters. Furthermore, Cd treatment caused severe disruption of chloroplast organization and reduced chlorophyll fluorescence compared to control. Both PDJ and Si treatments provided protective effects against Cd toxicity, improving chloroplast structure and fluorescence intensity. The combined PDJ + Si treatment showed the most effective protection, nearly restoring normal chloroplast organization (**Figure 5A**). Furthermore, Cd treatment caused significant effect on cell wall thickening and structural deformation compared to the control group. Both PDJ and Si treatments reduced Cd-induced cell wall alterations and helped maintain cellular integrity. The combined PDJ + Si treatment provided optimal protection, preserving near-normal cell wall structure and organization under Cd stress conditions (**Figure 5B**). These results demonstrate synergistic protective effects that maintain photosynthetic integrity and chlorophyll stability under Cd stress, indicating coordinated mechanisms for preserving cellular photosynthetic machinery.

### 3.3 Physiological resilience and hormonal regulation

**Figure 6:**
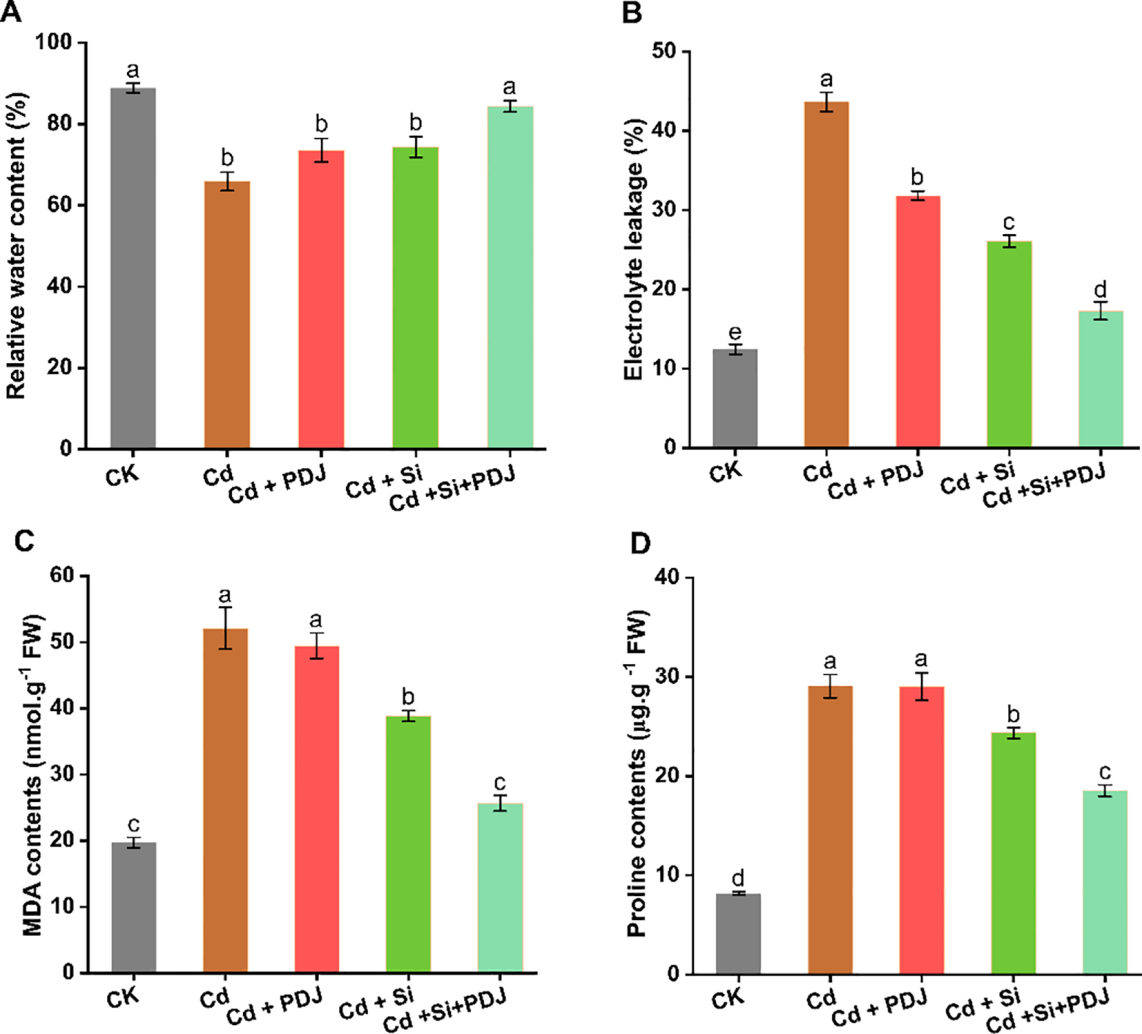
Effects of PDJ-Si application on cellular membrane stability and water balance regulation in rice seedlings exposed to Cd stress. Evaluated physiological indicators comprise: **(A)** Tissue water retention capacity, **(B)** Ion efflux from cells, **(C)** Malondialdehyde accumulation and **(D)** Proline biosynthesis levels under various treatment conditions. Data represent average measurements ± standard error (*n* = 6 tests/treatment). Data are presented as mean ± standard error. Different letters above bars indicate statistically significant difference between treatments (*p* < 0.05) using ANOVAs followed by *post-hoc* analysis.

**Figure 7:**
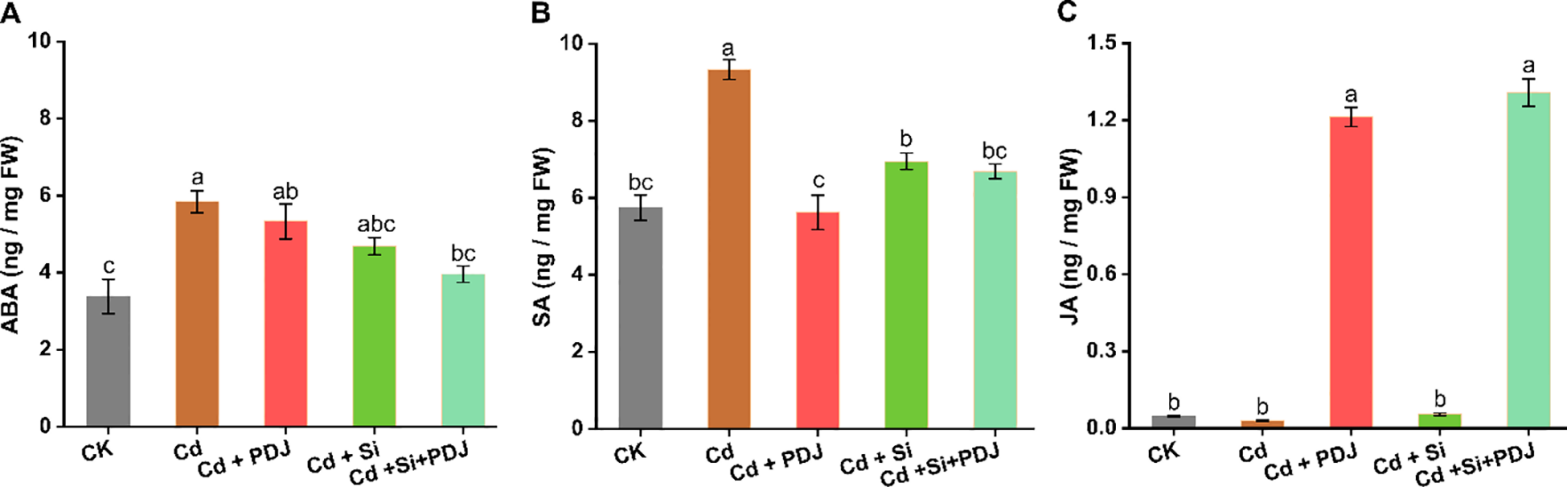
Influence of PDJ-Si application on plant hormone accumulation in rice seedlings under Cd stress. Concentrations of **(A)** ABA **(B)** SA and **(C)** JA measured across different treatment groups. Data are presented as mean ± standard error (*n* = 4 tests/treatment). Different letters above bars indicate statistically significant difference between treatments (*p* < 0.05) using ANOVAs followed by *post-hoc* analysis.

PDJ-Si co-treatment significantly improved membrane stability and osmotic regulation under Cd stress (**Figure 6**). RWC decreased from 92.3% (control) to 65-75% under Cd treatments, with Si+PDJ showing notable recovery to 82.1% (*F_4_,_29_* = 17.82, *p* < 0.001; **Figure 6A**). Electrolyte leakage increased under Cd stress (44.7% vs 13.2% in control), while Si+PDJ treatment reduced leakage to 18.4%, representing 58.8% improvement over Cd alone (*F_4_,_29_* = 191.9, *p* < 0.001; **Figure 6B**). MDA accumulation indicated severe oxidative stress under Cd exposure, with Si+PDJ treatment providing maximum protection (52.4% reduction; *F_4_,_29_* = 62.23, *p* < 0.001; **Figure 6C**). Proline content increased substantially under all Cd treatments, with Si treatments showing moderated accumulation (*F_4_,_29_* = 95.93, *p* < 0.001; **Figure 6D**). Phytohormone analysis revealed treatment-specific regulatory responses (**Figure 7**). ABA levels showed moderate elevation under Cd stress, with treatments maintaining levels within optimum range (*F_4_,_19_* = 8.64, *p* < 0.001; **Figure 7A**). Similarly, SA accumulation was most pronounced under Cd stress alone, while other treatment maintained moderate levels (*F_4_,_19_* = 24.32, *p* < 0.001; **Figure 7B**). JA responses showed consistent activation in PDJ-containing treatments, indicating coordinated stress signaling (*F_4_,_19_* = 515.13, *p* < 0.001; **Figure 7C**). These results demonstrate that PDJ-Si co-treatment could provide comprehensive protection through integrated membrane stabilization, oxidative stress mitigation and balanced hormonal regulations.

### 3.4 Expression patterns of genes regulating phytohormone induction, cd transport and detoxification

**Figure 8:**
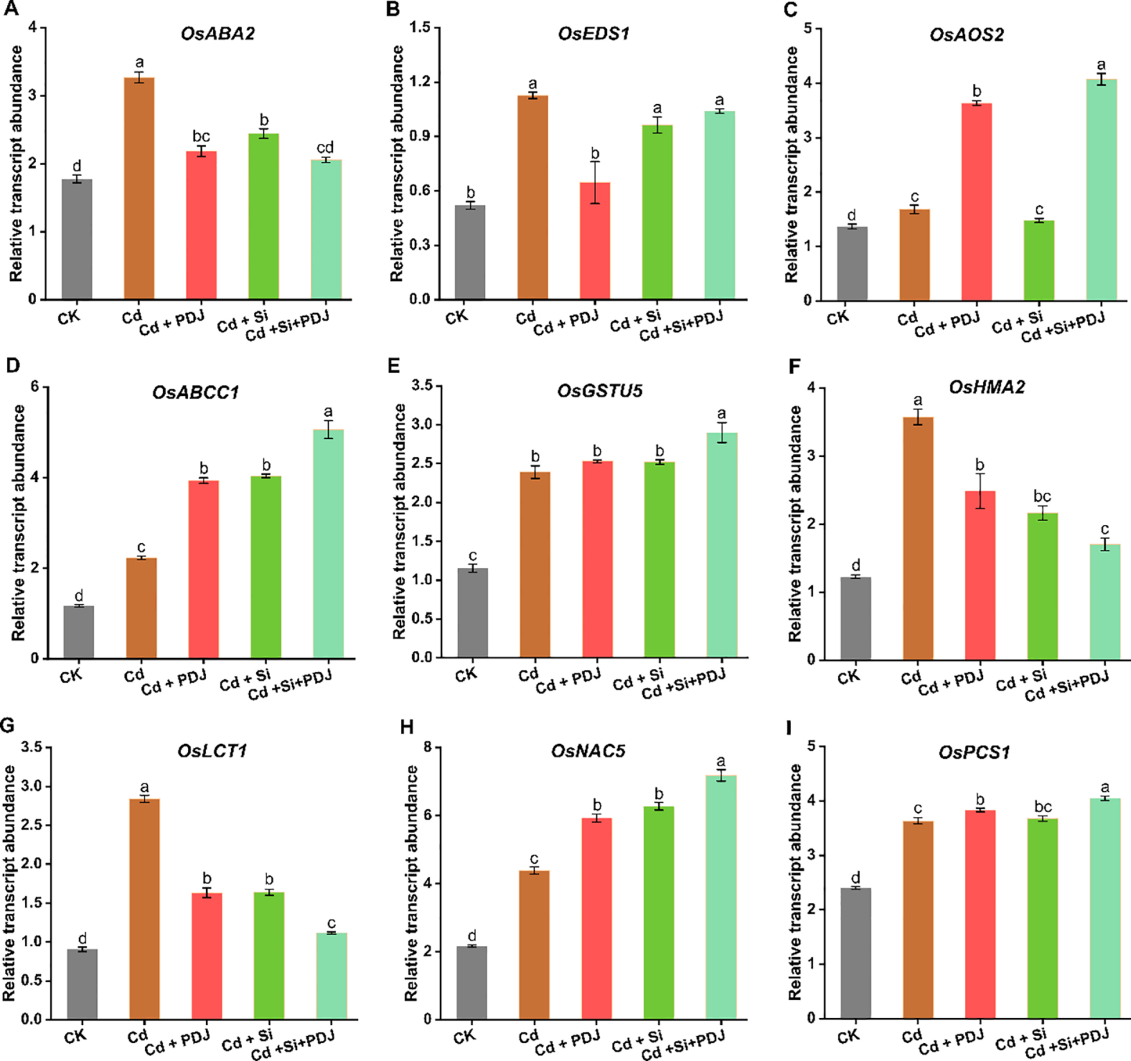
PDJ-Si treatment alters the expression genes regulating phytohormones induction, Cd transport and detoxification. **(A-C)** Relative transcript abundance of genes involved in hormone biosynthesis **(D, E)** stress response and **(F-I)** metal tolerance and detoxification in rice seedlings under different treatment conditions. Data are presented as mean ± standard error (*n* = 3 tests/treatment). Different letters above bars indicate statistically significant difference between treatments (*p* < 0.05) using ANOVAs followed by *post-hoc* analysis.

Gene expression analysis revealed that PDJ-Si co-treatment modulated key regulatory pathways involved in hormone biosynthesis, Cd transport and detoxification pathways (**Figure 8**). Hormone-related genes showed treatment-specific responses, with *OsABA2* expression increased under Cd stress and decreased in Cd+PDJ, Cd+Si and PDJ+Si treatment (F_4_,_24_ = 73.02, *p* < 0.001; **Figure 8A**). Similarly, *OsEDS1* expression was significantly enhanced under Cd stress but decreased to near-control levels with PDJ treatments alone (*F_4_,_24_* = 21.11, *p* < 0.001; **Figure 8B**). *OsAOS2* showed moderate upregulation following Cd treatments, with Cd+PDJ or Si+PDJ achieving highest expression (F_4_,_24_ = 375.08, *p* < 0.001; **Figure 8C**). Stress response genes *OsABCC1* and *OsGSTU5* exhibited substantial upregulation under all Cd treatments, with Si+PDJ treatment showing maximum induction (*F_4_,_24_* = 256.21, *p* < 0.001 and *F_4_,_24_* = 83.9, *p* < 0.001; **Figures 8D, E**). HMs tolerance genes *OsHMA2* and *OsNAC5* displayed contrasting patterns, with *OsHMA2* showing reduced expression in Si or PDJ treatments while *OsNAC5* maintained consistent upregulation (*F_4_,_24_* = 39.89, *p* < 0.001 and *F_4_,_24_* = 291.32, *p* < 0.001; **Figures 8F, H**). Notably, *OsLCT1* expression was dramatically induced in Cd treatment but suppressed in Si, PDJ and Si+PDJ treatments, while *OsPCS1* showed uniform upregulation across all Cd treatments and Si+PDJ induced higher expression (*F_4_,_24_* = 319, *p* < 0.001and *F_4_,_24_* = 230.82, *p* < 0.001; **Figures 8G, I**). These molecular responses indicate that PDJ-Si treatment orchestrates Cd tolerance through coordinated regulation of hormone biosynthesis, stress response activation and metal detoxification pathways.

### 3.5 Integrated correlation matrix reveals stress-responsive networks

**Figure 9:**
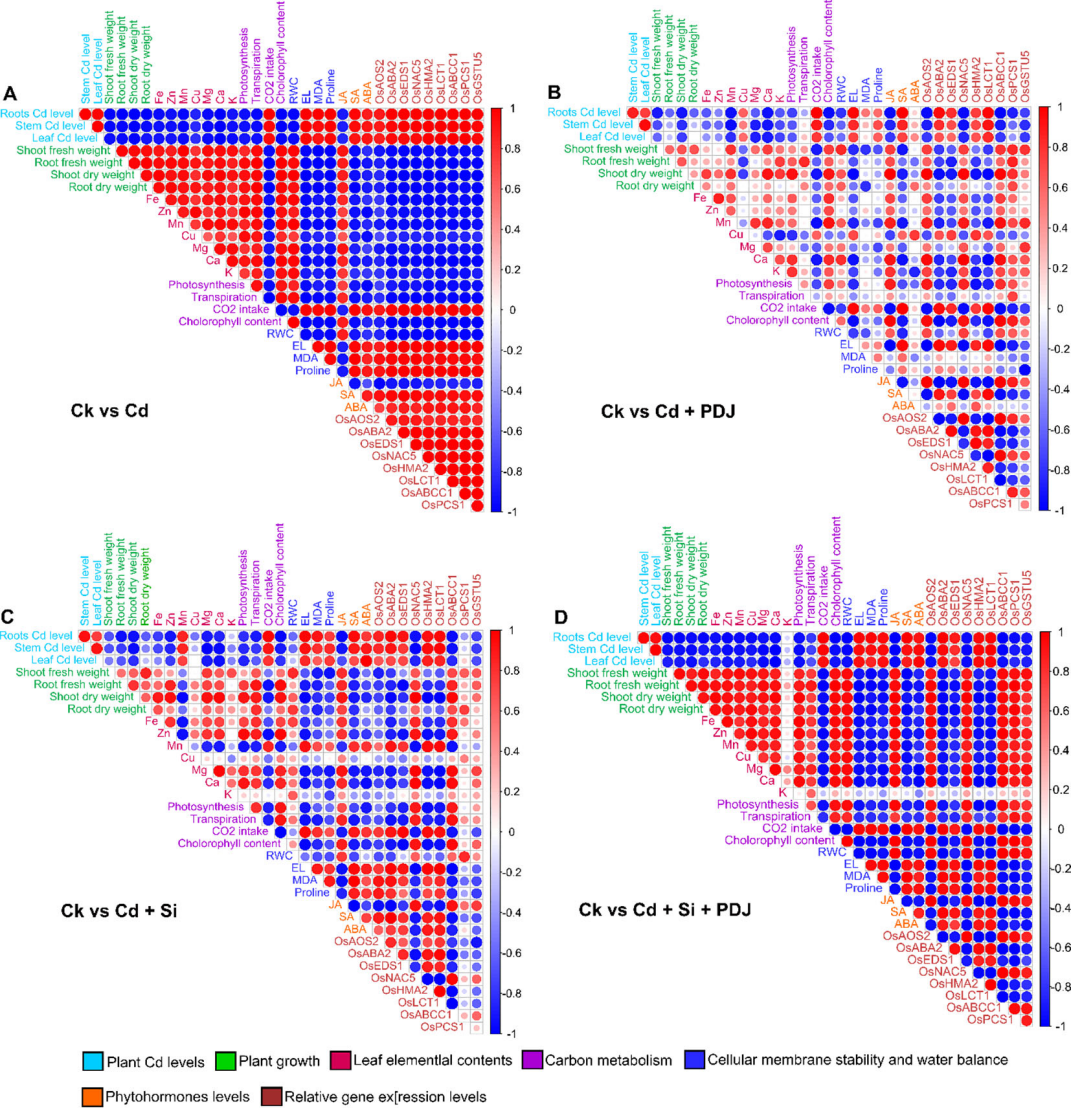
Integrated correlation matrices illustrating the interactive effects of PDJ and Si on physiological, biochemical and molecular responses of rice seedlings under cadmium stress. Each heatmap represents Pearson’s correlation coefficients among all measured parameters under four conditions: **(A)** Cd stress alone, **(B)** Cd + PDJ, **(C)** Cd + Si and **(D)** Cd + PDJ + Si. Blue and red circles denote negative-positive correlation, with color intensities and circle size proportional to the correlation strength (*p* < 0.05).

Comprehensive correlation analyses (**Figure 9**) revealed distinct interaction networks among physiological, biochemical and molecular parameters under different treatment regimes. In Cd-stressed plants, strong positive correlations were evident among Cd accumulation in roots, stems and leaves, reflecting systemic metal translocation. Elevated Cd levels showed significant negative correlations with photosynthetic parameters (Pn, Gs, E, Ci) and biomass indices (root/shoot weights), confirming the inhibitory effects of Cd on growth and photosynthetic efficiency. The concurrent positive relationships between Cd and oxidative stress markers (MDA, Proline) and ABA biosynthetic genes (*OsABA2*, *OsNCED3*) indicated stress-induced activation of lipid peroxidation and ABA signaling (**Figure 9A**). Under PDJ treatment alone the Cd–photosynthesis antagonism was moderately alleviated, as evidenced by partial restoration of positive correlations between photosynthetic efficiency and growth parameters. PDJ markedly enhanced the coordination between antioxidant activity (MDA, Proline as osmoprotectant) and the upregulation of JA-related genes (*OsAOS2*, *OsAOC*, OsLOX2), suggesting PDJ-driven activation of jasmonate-mediated defense pathways (**Figure 9B**).

Si supplementation established a distinct correlation pattern dominated by strong negative linkages between tissue Cd levels and Si-accumulation-dependent physiological attributes. Si application improved root Cd sequestration while maintaining positive correlations among chlorophyll content, photosynthetic rate and nutrient elements (Fe, Mn, K), reflecting structural and ionic stabilization of cellular metabolism under stress (**Figure 9C**).

Remarkably, the PDJ–Si co-treatment exhibited a harmonized network wherein photosynthetic parameters, RWC and biomass indices displayed strong positive inter-correlations with antioxidant and hormone-related traits. Root Cd retention correlated positively with detoxification and transport genes (*OsABCC1*, *OsPCS1*, *OsGSTU5*, *OsHMA2*) and negatively with leaf Cd content, signifying efficient restriction of Cd translocation. Furthermore, balanced associations among SA, JA and ABA pathways (*OsEDS1*, *OsAOS2*, *OsABA2*) under PDJ–Si co-treatment confirmed multi-hormonal coordination that underpins enhanced detoxification, redox homeostasis, and stress resilience (**Figure 9D**). Collectively, the correlation matrices demonstrate that the PDJ–Si synergy re-establishes functional integration across physiological, biochemical and transcriptional levels, optimizing Cd detoxification and maintaining metabolic coherence in rice under metal stress.

## 4 Discussion

### 4.1 Organ-specific Cd translocation and sequestration

The increasing global concerns over Cd contamination in agricultural soil necessitates innovative approaches to safeguard crop productivity and food-security (Huang et al., 2022; Wang et al., 2019b). Current study investigates the synergistic potential of PDJ and Si supplementation in mitigating Cd toxicity in rice, revealing novel protective mechanisms that operate through coordinated physiological, molecular and biochemical pathways. Our findings demonstrate that the combined PDJ+Si treatment represents a breakthrough in phytoremediation strategies, achieving superior protection compared to individual PDJ or Si treatments through previously uncharacterized synergistic interactions.

Our results reveal a distinctive organ-specific Cd accumulation pattern, with roots serving as the primary sequestration site, followed by stems and leaves in decreasing order. This gradient distribution pattern aligns with established understanding of plant HMs tolerance mechanisms, where root tissues function as first line of defense upon metal toxicity (Ghuge et al., 2023). For example, Si application has been known to ameliorate Cd stress through altering subcellular distribution, enhanced Cd retention on root cell wall and restricting transportation to shoots in rice seedlings (Wei et al., 2021). However, in our study the synergistic PDJ-Si treatment achieved unprecedented Cd reduction, compared to Cd-alone control, significantly outperforming individual PDJ or Si treatments. This improved efficacy suggests that PDJ or Si operate through complementary mechanisms rather than additive affects: Si likely reinforce root cell wall structure and restricts Cd transport into stem, while PDJ enhances cellular detoxification processes as well as metal sequestration within root vacuoles, preventing translocation to sensitive aerial tissues. For instance, synthetic analogue of JA, such as MeJA can reduce Cd stress in wheat crop (Repkina et al., 2023). Naturally occurring jasmonates (JAs), which are lipid-derived compounds, play important functions in promoting crops health, particularly under heavy-metals stress (Raza et al., 2021). Additionally, Si can enhance the growth and biomass accumulation of rice seedlings, alleviate Cd toxicity, and protect roots from damage (Kim et al., 2014). Notably, PDJ, a more stable analog of JA, can further enhance heavy metal stress tolerance, especially when applied in combination with elements like Si. The combined treatment of PDJ-Si operates through complementary mechanisms: PDJ likely enhances cellular detoxification processes through bioactive compounds, while Si strengthens structural barriers and modifies metal transport pathways, collectively restricting Cd absorption to roots tissues and preventing systemic accumulation in edible plants parts. The superior efficacy of the combined treatment indicates novel molecular interactions that amplify individual protective effects, representing a significant advancement in understanding plant-based metal stress mitigation (Labudda et al., 2022).

### 4.2 Leaf elemental homeostasis

The restoration of leaf elements homeostasis under combined PDJ-Si treatment reveals previously unrecognized mechanisms of nutrient recovery during metals stress (Ghori et al., 2019; Umar et al., 2025). Cd exposure systematically depleted all elements, with Fe showing the most severe decline, followed by K and Mn. This widespread elements disruption reflects Cd interference with membrane transport systems and enzymatic processes involved in nutrient uptake (Pasricha et al., 2021; Qin et al., 2020). Remarkably, the combined treatment achieved substantial recovery of elements concentrations compared to control levels, substantially exceeding individual treatment efficacy. This exceptional mineral restoration suggests that PDJ-Si treatment operates through multiple pathways: Si may enhance membrane integrity and selective permeability by stabilizing lipid bilayers and restoring ion channel function (Rahman et al., 2017), while PDJ compounds could modulate transporter gene expression or chelate Cd ions that that competitively inhibit nutrient uptake (Takahashi et al., 2021), thereby alleviating the metabolic stress imposed by Cd on nutrient acquisition system. The differential recovery patterns across elements indicate element-specific protection mechanisms that warrant further investigation into the molecular basis of these interactions.

### 4.3 Photosynthetic recovery and chloroplast protection

Photosynthetic recovery under PDJ+Si treatment demonstrates sophisticated protection of the photosynthetic apparatus that extends beyond simple structural preservation. Cd stress severely impaired all photosynthetic parameters, with substantial reductions in net photosynthetic rates and chlorophyll contents (Parmar et al., 2013), reflecting disruption of both photochemical reactions and pigment stability. However, the combined treatment of PDJ+Si achieved remarkable recovery, restoring photosynthetic parameters to levels approaching untreated control. This comprehensive photosynthetic restoration indicates protection of multiple photosynthetic components: Si likely stabilizes thylakoid membranes and maintains chloroplast structural integrity by incorporating into membrane lipids and reducing ROS damage (Rastogi et al., 2021; Wang et al., 2019c), while PDJ may provide antioxidant protection against photosystem damage through direct scavenging of free radicals or upregulation of antioxidant enzymes. These coordinated recovery of gas exchange parameters and chlorophyll content suggests that the PDJ+Si treatments preserve both the biochemical machinery and physical infrastructure of photosynthesis, representing a holistic protective mechanism.

### 4.4 Membrane stability and osmotic adjustment

The physiological resilience mechanisms revealed by PDJ+Si treatment unveil novel insights into stress adaptation strategies. The dramatic improvement in membranes stability, evidenced by reduced electrolytes leakages and decreased lipids peroxidation, indicates comprehensive membrane protection (Anwar et al., 2023; El-Mahrouk et al., 2024). These improvements suggest that Si incorporation into cell walls and membrane enhances structural stability against oxidative damage, while PDJ-derived compounds provide biochemical protection through antioxidant mechanisms (Li et al., 2024). Concurrently, the maintenance of relative water content demonstrates superior osmoregulatory capacity of rice seedlings. Furthermore, the proline accumulation in PDJ-Si treatments remains proportionate to stress severity, indicating optimized osmotic adjustment without excessive energy expenditure, suggesting efficient stress adaptation rather than merely stress tolerance.

### 4.5 Phytohormonal coordination of Cd tolerance

Phytohormone regulation under PDJ+Si treatment reveals sophisticated signaling networks that coordinate stress responses and growth maintenance (Bali et al., 2019). The treatment-specific activation of salicylic acid in PDJ treatments coupled with consistent JA elevation indicates coordinated activation of defense pathways that suppress Cd toxicity (Yoshida et al., 2021). Simultaneously, the maintenance of ABA within optimal ranges demonstrates balanced stress signaling that avoids growth inhibition (Khan et al., 2021), indicating that PDJ compounds may directly modulate hormone biosynthesis or cellular sensitivity to hormonal signals, while Si influences stress perception and signal transduction pathways.

### 4.6 Molecular regulation of Cd tolerance

Molecular analysis reveals that PDJ+Si treatment orchestrates Cd tolerance through coordinated regulation of hormone biosynthesis, metal transport and detoxification pathways (Küçükrecep et al., 2024). The substantial induction of stress response genes *OsABCC1* and *OsGSTU5* indicates enhanced cellular detoxification capacity through both vacuolar sequestration and glutathione-dependent conjugation pathways (Yang et al., 2023), while the contrasting regulation of metal transport genes *OsHMA2* and *OsLCT1* suggests selective modulation of Cd uptake and xylem loading restricting root-to-shoot translocation (Tian et al., 2019). For example, Si alone has been found to decreased Cd accumulation shoots and roots, which is regulated by the transporter genes responsible for Cd uptake and translocation in rice (Feng Shao et al., 2017). Furthermore, the particular and uniform upregulation of *OsPCS1* across different treatments, with maximum expression in combined treatment, indicating enhanced phytochelatin synthesis for HMs sequestration and demonstrating that this core detoxification pathways is amplified by synergistic PDJ+Si treatment (Yamazaki et al., 2018). Moreover, the treatments-specific expression patterns of hormone biosynthesis genes (*OsABA2*, *OsEDS1*, *OsAOS2*) demonstrate molecular fine-tuning of Cd-stress responses that align with the observed phytohormones profiles (Li et al., 2023; Yoshida et al., 2021), indicating PDJ and Si influence distinct but complementary gene regulatory networks operating at transcriptional level. This systems-level protection explains the superior efficacy of combined treatments and suggests that effective stress mitigation requires addressing multiple physiological targets simultaneously.

### 4.7 Study limitations

While our results demonstrate the synergistic benefits of PDJ and Si treatment on plant physiology and gene expression, it is important to note that these findings were obtained under controlled greenhouse conditions. Field conditions involve greater environmental variability and diverse soil microbial communities that may alter treatment efficacies, suggesting that future work should validate these findings in natural settings. Additionally, longitudinal studies incorporating genomics and metabolomics approaches would provide deeper mechanistic insights into the sustained effects of this treatment combination over extended growth periods.

## 6 Conclusion

This study demonstrate that the combined application of PDJ-Si offers a more robust protective strategy against Cd stress in rice that either component alone. Their synergistic applications reduce Cd accumulation, restored mineral homeostasis, improve photosynthetic capacity, stabilized membranes, optimized osmotic adjustment and regulate key hormonal and transcriptional pathways. The coordinated improvement across traits indicate that Cd tolerance arises from simultaneous reinforcement of multiple physiological and molecular processes, rather than from a single dominant mechanism. By identifying a synergistic interaction between PDJ and Si, this study highlights a multi-target approach that may be more effective than conventional single–agent strategies for managing HMs stress. However, these findings remain constrained by short-term measurement and the absence of whole–plant life cycle assessment. Future studies, examining long term performance grain safety and underlying biochemical interactions will determine the practical application of this synergy in contaminated agricultural system.

## Data Availability

The original contributions presented in the study are included in the article/**Supplementary Material**, further inquiries can be directed to the corresponding author.
