# Supplementary material for: Prohydrojasmonate–silicon synergy enhances cadmium detoxification and stress tolerance in rice, Oryza sativa L
Source: Front Plant Sci. 2026 Jan 19;16:1731423. doi: 10.3389/fpls.2025.1731423 (PMC12862827; doi:10.3389/fpls.2025.1731423)
Supplement: Supplementary file 1 [file Table1.docx]

**Supporting Information**

**Prohydrojasmonate–silicon synergy enhances cadmium detoxification and stress tolerance in rice, *Oryza sativa* L.**

**Salem M. AL-Amri**^*^

The following Supporting Information is available for this article:

**Table 1.** Primer sequences used for RT-PCR study.

| Gene name | Forward primer (5’-3’) | Reverse primer (5’-3’) | References |
| --- | --- | --- | --- |
| *OsAOS1* | AGC TAGCTA GAA GAG AGT TAG C | ACTCGA AGT ACT TGT CCT GC- | (Mei et al., 2006) |
| *OsABA2* | TCATTCATACACAGCAACCAAGCAT | CTTCACCACCAACAAAGGCGAAA | (Zhang et al., 2022) |
| *OsEDS1* | AAGCAAAGCATGATGCAAGA | TCAGAGACTTGAGGGCTTCC | (Wu et al., 2024) |
| *OsNAC5* | GCACGAGTACCGCCTC | ACCCTGAGGGCGTTGTG | (Li et al., 2024) |
| *OsHMA2* | CATAGTGAAGCTGCCTGAGATC | GATCAAACGCATAGCAGCATCG | (Hu et al., 2024) |
| *OsLCT1* | GAGTTCTTCGTCAGAGCTAC | CAGTGCTGGATGACGAATTG | (Xia et al., 2023) |
| *OsABCC1* | AACAGTGGCTTATGTTCCTCAAG | AACTCCTCTTTCTCCAATCTCTG | (Song et al., 2014) |
| *OsPCS1* | GACTTCCGCCACCATCTC | CTTTTGCAAGGACACCAGCC | (Hayashi et al., 2017) |
| *OsGSTU5* | CATCCTCGAGTACATCGACGAGACATG | CTGAGCTTCCAGAGGCGTGTCT | (Tiwari et al., 2022) |

**References**

Hayashi, S., Kuramata, M., Abe, T., Takagi, H., Ozawa, K., Ishikawa, S., 2017. Phytochelatin synthase OsPCS1 plays a crucial role in reducing arsenic levels in rice grains. The Plant Journal 91, 840-848.

Hu, S., Chen, J., Wang, H., Ji, E., Su, X., Zhu, M., Xiang, X., Gong, L., Zhou, Q., Xiao, X., Wu, G., Zha, H., 2024. The transcription factor OsNAC5 regulates cadmium accumulation in rice. Ecotoxicology and Environmental Safety 285, 117102.

Li, R., Song, Y., Wang, X., Zheng, C., Liu, B., Zhang, H., Ke, J., Wu, X., Wu, L., Yang, R., Jiang, M., 2024. OsNAC5 orchestrates OsABI5 to fine-tune cold tolerance in rice. Journal of Integrative Plant Biology 66, 660-682.

Mei, C., Qi, M., Sheng, G., Yang, Y., 2006. Inducible Overexpression of a Rice Allene Oxide Synthase Gene Increases the Endogenous Jasmonic Acid Level, PR Gene Expression, and Host Resistance to Fungal Infection. Molecular Plant-Microbe Interactions® 19, 1127-1137.

Song, W.Y., Yamaki, T., Yamaji, N., Ko, D., Jung, K.H., Fujii-Kashino, M., An, G., Martinoia, E., Lee, Y., Ma, J.F., 2014. A rice ABC transporter, OsABCC1, reduces arsenic accumulation in the grain. Proc Natl Acad Sci U S A 111, 15699-15704.

Tiwari, M., Kidwai, M., Dutta, P., Narayan, S., Gautam, N., Chawda, K., Shirke, P.A., Mishra, A.K., Chakrabarty, D., 2022. A tau class glutathione-S-transferase (OsGSTU5) confers tolerance against arsenic toxicity in rice by accumulating more arsenic in root. Journal of Hazardous Materials 426, 128100.

Wu, Y., Xu, W., Zhao, G., Lei, Z., Li, K., Liu, J., Huang, S., Wang, J., Zhong, X., Yin, X., Wang, Y., Zhang, H., He, Y., Ye, Z., Meng, Y., Chang, X., Lin, H., Wang, X., Gao, Y., Chai, J., Parker, J.E., Deng, Y., Zhang, Y., Gao, M., He, Z., 2024. A canonical protein complex controls immune homeostasis and multipathogen resistance. Science 386, 1405-1412.

Xia, R., Zhou, J., Cui, H., Liang, J., Liu, Q., Zhou, J., 2023. Nodes play a major role in cadmium (Cd) storage and redistribution in low-Cd-accumulating rice (Oryza sativa L.) cultivars. Science of The Total Environment 859, 160436.

Zhang, G., Shen, T., Ren, N., Jiang, M., 2022. Phosphorylation of OsABA2 at Ser197 by OsMPK1 regulates abscisic acid biosynthesis in rice. Biochemical and Biophysical Research Communications 586, 68-73.
